# Clinical characteristics and outcomes of a patient population with atypical hemolytic uremic syndrome and malignant hypertension: analysis from the Global aHUS registry

**DOI:** 10.1007/s40620-022-01465-z

**Published:** 2022-09-24

**Authors:** Jean-Michel Halimi, Imad Al-Dakkak, Katerina Anokhina, Gianluigi Ardissino, Christoph Licht, Wai H. Lim, Annick Massart, Franz Schaefer, Johan Vande Walle, Eric Rondeau

**Affiliations:** 1 Service de Néphrologie-Hypertension Artérielle, Dialyses, Transplantation Rénale, CHRU Tours, Tours, France; 2 University of Tours, Equipe d’Accueil 4245 (EA4245), Tours, France; 3 Alexion, AstraZeneca Rare Disease, Boston, MA USA; 4 Center for HUS Control, Prevention and Management, Fondazione IRCCS Ca’ Granda Ospedale Maggiore Policlinico, Milan, Italy; 5 Division of Nephrology, The Hospital for Sick Children, Toronto, ON Canada; 6 Department of Renal Medicine, Sir Charles Gairdner Hospital, Perth, Australia; 7 Medical School, University of Western Australia, Perth, Australia; 8 Department of Nephrology and Hypertension, Antwerp University Hospital, Edegem, Belgium; 9 Division of Pediatric Nephrology, Heidelberg University Hospital, Heidelberg, Germany; 10 Department of Internal Medicine and Pediatrics, Ghent University Hospital, Ghent, Belgium; 11 Urgences Néphrologiques et Transplantation Rénale, Hôpital Tenon, Paris, France

**Keywords:** Complement, Eculizumab, Hemolytic uremic syndrome, Malignant hypertension

## Abstract

**Introduction:**

Atypical hemolytic uremic syndrome (aHUS) is a rare form of thrombotic microangiopathy (TMA) often caused by alternative complement dysregulation. Patients with aHUS can present with malignant hypertension (MHT), which may also cause TMA.

**Methods:**

This analysis of the Global aHUS Registry (NCT01522183) assessed demographics and clinical characteristics in eculizumab-treated and not-treated patients with aHUS, with (*n* = 71) and without (*n* = 1026) malignant hypertension, to further elucidate the potential relationship between aHUS and malignant hypertension.

**Results:**

While demographics were similar, patients with aHUS + malignant hypertension had an increased need for renal replacement therapy, including kidney transplantation (47% vs 32%), and more pathogenic variants/anti-complement factor H antibodies (56% vs 37%) than those without malignant hypertension. Not-treated patients with malignant hypertension had the highest incidence of variants/antibodies (65%) and a greater need for kidney transplantation than treated patients with malignant hypertension (65% vs none). In a multivariate analysis, the risk of end-stage kidney disease or death was similar between not-treated patients irrespective of malignant hypertension and was significantly reduced in treated vs not-treated patients with aHUS + malignant hypertension (adjusted HR (95% CI), 0.11 [0.01–0.87], *P* = 0.036).

**Conclusions:**

These results confirm the high severity and poor prognosis of untreated aHUS and suggest that eculizumab is effective in patients with aHUS ± malignant hypertension. Furthermore, these data highlight the importance of accurate, timely diagnosis and treatment in these populations and support consideration of aHUS in patients with malignant hypertension and TMA.

**Trial registration details:**

Atypical Hemolytic-Uremic Syndrome (aHUS) Registry.

Registry number: NCT01522183 (first listed 31st January, 2012; start date 30th April, 2012).

**Graphical abstract:**

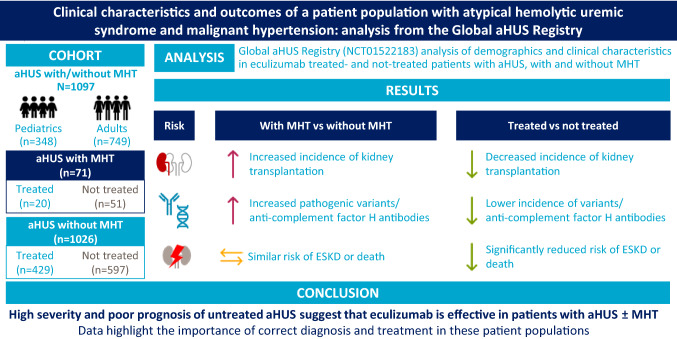

**Supplementary Information:**

The online version contains supplementary material available at 10.1007/s40620-022-01465-z.

## Introduction

Atypical hemolytic uremic syndrome (aHUS) is a rare form of thrombotic microangiopathy (TMA) typically caused by alternative complement pathway dysregulation, that is often classified as a complement-mediated TMA (CM-TMA) [1–4]. aHUS is characterized by thrombocytopenia, microangiopathic hemolytic anemia, and acute kidney injury and can also present as progressive kidney damage, or as extrarenal manifestations resulting in damage to other organs [5–7]. Another condition that can result in TMA is malignant hypertension (MHT), a severe form of arterial hypertension traditionally diagnosed by high blood pressure (diastolic pressure > 120 mmHg) with papilledema/hypertensive retinopathy [8–12]. More recent experience has emphasized the role of multi-organ involvement/damage in the diagnosis and prognosis of MHT, and MHT with multi-organ involvement has also been referred to as hypertensive emergency [10, 13]. The kidneys are frequently affected in patients with MHT, and patients often present with elevated serum creatinine, proteinuria, hemolysis, low platelet count, and kidney failure, all of which are also key markers of TMA [10, 14]. Further, complement dysregulation has also been implicated in patients with hypertension-associated TMA, with one study finding that 87.5% of patient serum samples induced formation of abnormal C5b-9 on microvascular endothelial cells in vitro*.* This has previously been proposed as a highly specific assessment of complement dysregulation/activation in patients with aHUS [15].

Previous studies have suggested that aHUS and MHT are common comorbid conditions, although their precise relationship has often been unclear [16–19]. Recent evidence suggests that while MHT is highly prevalent in patients with aHUS, among all cases of MHT, aHUS remains a marginal cause. There is also evidence of direct associations between MHT and development of TMA [8, 9, 13, 20]. The interplay/overlap between these conditions means that establishing causality is often extremely difficult. Despite the difficulties associated with differentiating between MHT and aHUS, establishing a clear and correct diagnosis is extremely important as the underlying mechanisms and treatment choices differ significantly. The current standard of care in patients diagnosed with aHUS is complement C5 inhibitor therapy, while patients presenting with MHT will typically be treated with blood pressure lowering medications [21, 22]. Due to the substantially different pathophysiological mechanisms underlying these conditions, delays in diagnosis and sub-optimal treatment regimens can have considerable, negative effects on patient outcomes. Finally, it is presently unknown whether the complement C5 inhibitor eculizumab is effective in treating patients with aHUS and MHT.

Using data from the Global aHUS Registry, the largest registry of real-world data relating to patients with aHUS, this analysis characterized pediatric and adult patients with aHUS, both with and without MHT, who were either treated or not treated with eculizumab. This study explored the baseline characteristics of these patient groups and assessed the risk of reaching the composite endpoint of end-stage kidney disease (ESKD) or death. Clinical characteristics and outcomes are also presented by adult and pediatric designation.

## Methods

**Fig. 1:**
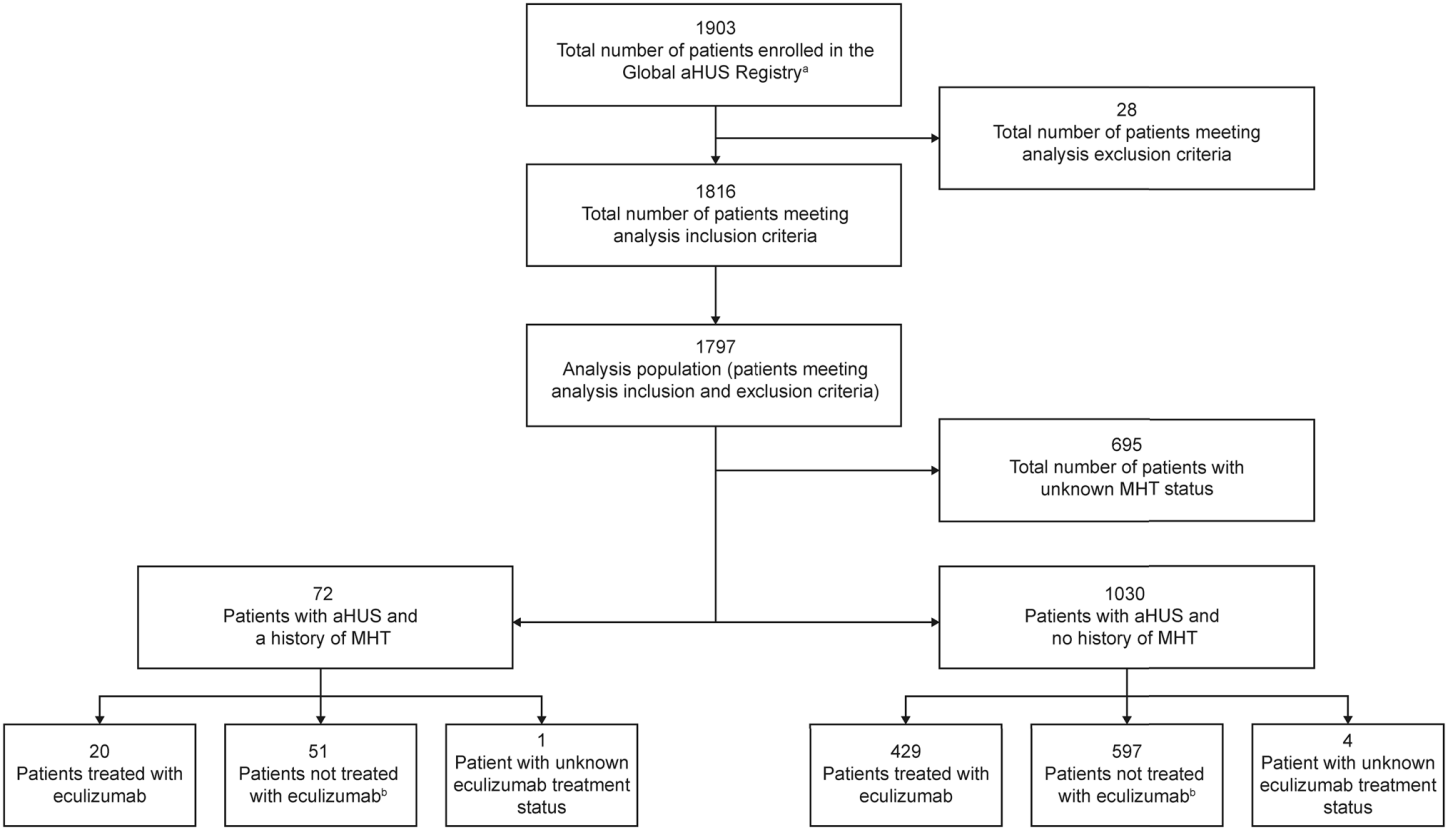
Patient disposition. ^a^Includes all patients enrolled in the Global aHUS Registry from April 2012 to 26 October, 2020; ^b^Not-treated patients included any patients who were never treated with eculizumab; who received eculizumab after reaching ESKD, defined as kidney transplantation or chronic maintenance dialysis; or who received eculizumab up to and including 1 month prior to kidney transplantation. *aHUS* atypical hemolytic uremic syndrome, *MHT* malignant hypertension

This retrospective analysis utilized data from the Global aHUS Registry (NCT01522183), an observational, non-interventional, multicenter registry that retrospectively and prospectively collects demographic information, natural history data, and treatment outcomes of patients with aHUS. The registry methodology and initial patient characteristics have previously been reported [23]. This analysis included patients enrolled into the registry from April 2012 until 26 October, 2020 [23]. Patients were included if they were enrolled in the registry and were followed up for ≥ 90 days after initial aHUS presentation or diagnosis date. aHUS was diagnosed locally, with no central registry definition of aHUS used. Patients in the MHT cohort were also required to have a recorded diagnosis of MHT, as defined by the local registry investigator/treating physician, and applied criteria usually included diastolic blood pressure > 120 mmHg, alongside papilledema, retinopathy and/or exudates. No definition of severe hypertension was available within the registry. Patients were excluded from this analysis if they withdrew consent from the registry or discontinued eculizumab due to a revised diagnosis of any condition other than aHUS. To assess the effects of eculizumab on outcomes, patients were defined as either treated or not-treated. Patients not treated with eculizumab included any patients who were never treated with eculizumab, or who received eculizumab after reaching ESKD (defined as kidney transplantation or chronic maintenance dialysis), or who received eculizumab up to and including one month prior to kidney transplantation. No minimum duration of eculizumab administration was required for inclusion in the treated group. Patient disposition for this analysis is presented in Fig. 1.

The following variables were extracted for analysis; age at aHUS diagnosis, sex, time to eculizumab initiation, family history of aHUS, timing of MHT diagnosis (related to the time of initial aHUS presentation), new extra-renal manifestations of aHUS not present at initial diagnosis (number and organ system), pathogenic genetic variant status and presence of autoantibodies to complement factor H, triggering conditions other than MHT, kidney transplant status, and baseline serum creatinine, platelet counts and lactate dehydrogenase levels. Baseline was defined as the closest value to aHUS onset in either direction. The primary outcome of interest was the composite endpoint of time to ESKD or death. The variables and primary endpoint were stratified by treatment status, MHT status, and age group (pediatric [< 18 years] vs adult).

### Statistical analysis

Continuous data were summarized as median (min, max), while categorical data were summarized as number of patients (%). Laboratory parameters were presented using both number of patients with available data (%) and median (min, max) for values. No formal statistical comparisons were performed on baseline characteristics data. Kaplan–Meier survival plots were generated for the composite endpoint, and hazard ratios (HRs) were calculated using Cox regression analysis. Both unadjusted and adjusted HRs and 95% confidence intervals are reported. HRs for the comparison of treated vs not-treated patients with aHUS and MHT were adjusted for plasma exchange/plasma infusion at the time of initial TMA, dialysis at the time of initial TMA, and the presence of any pathogenic genetic variants or anti-CFH antibodies. HRs for the comparison of not-treated patients with aHUS with vs without MHT were adjusted for age at initial onset of aHUS, sex, and the presence of any pathogenic genetic variants or anti-CFH antibodies. For assessment of the composite endpoint, propensity matching by age at initial onset of aHUS, sex, and presence of pathogenic genetic variants was performed. Additionally, only those patients with recorded genetic testing results had their genetic data included in the analysis. Any missing data were excluded from this analysis.

## Results

### Patient disposition

Patient disposition is presented in Fig. 1. At the time of this analysis, a total of 1903 patients were enrolled in the Global aHUS Registry. Following application of the inclusion and exclusion criteria, 1797 of the 1903 patients were eligible for this study. A further 695 patients were excluded due to unknown MHT status and five due to unknown eculizumab treatment status (1 with MHT, 4 without MHT). This analysis therefore included 1097 patients; 71 presenting with both aHUS and MHT (20 treated and 51 not treated with eculizumab) and 1026 presenting with aHUS without MHT (429 treated and 597 not treated with eculizumab).

Overall, 20 (28%) patients with aHUS and MHT were treated with eculizumab, compared to 429 (42%) patients without MHT. Of the 72 patients with aHUS and MHT, 23 (32%) had a recorded onset of aHUS prior to 2011, while of the 1030 patients with aHUS without MHT, 323 (31%) had a recorded onset of aHUS prior to 2011. Eculizumab was granted marketing authorization in 2011.

### Patient demographics

Key patient demographics are presented in Table 1 and patient demographics stratified by age group are presented in Supplementary Table S1. Age at aHUS diagnosis, sex, and family history of aHUS were all similar between patients with aHUS both with and without MHT, irrespective of treatment status. Patients with aHUS and MHT had a slight numerical increase in the percentage of new extra-renal manifestations of aHUS across all organ systems. Genetic screening for at least one pathogenic complement variant was conducted in 61 (86%) patients with aHUS and MHT, and in 742 (72%) patients with aHUS without MHT. Of these, 34 (48%) with aHUS and MHT had their results recorded in the registry, compared to 300 (29%) patients without MHT. Testing for anti-CFH antibodies was performed in 11 (16%) patients with aHUS and MHT and in 91 (9%) patients with aHUS without MHT. Among patients whose genetic screening results were entered in the registry database, those with aHUS and MHT had a higher proportion of pathogenic genetic variants or anti-CFH antibodies compared to aHUS patients without MHT (40 [56%] vs 382 [37%]). Further, patients with aHUS and MHT who were not treated with eculizumab were found to have a much higher proportion of pathogenic genetic variants or anti-CFH antibodies (33 [65%]) than those with aHUS and MHT who were treated with eculizumab (7 [35%]), or those with aHUS without MHT regardless of treatment status (treated, 152 [35%], not-treated, 230 [39%]).

**Table 1:** Demographics of patients with aHUS and comorbid MHT stratified by treatment status

Characteristics	aHUS with MHT			aHUS without MHT		
	Treated (*n* = 20)	Not-treated^a^ (*n* = 51)	All (*n* = 71)	Treated (*n* = 429)	Not-treated^a^ (*n* = 597)	All (*n* = 1026)
Age at aHUS diagnosis [years], median (min, max)	32.5 (0.5, 63.1)	26.0 (0.7, 68.2)	28.1 (0.5, 68.2)	29.70 (0.02, 90.63)	31.10 (0.003, 84.16)	30.36 (0.003, 90.63)
Sex [F], *n* (%)	11.0 (55.0)	32.0 (62.7)	43 (60.6)	279 (65.0)	345 (57.8)	624 (60.8)
Time between aHUS onset and eculizumab initiation [months], median (Q1, Q3)	1.0 (0.6, 3.2)	N/A	1.0 (0.6, 3.2)	0.7 (0.3, 3.6)	N/A	0.7 (0.3, 3.6)
Total duration of eculizumab treatment [months], n, median (min, max)	20, 33.0 (0.2, 71.6)	31, 37.1 (0.1, 110.9)	51, 33.9 (0.1, 110.9)	419, 22.0 (0.03, 145.3)	286, 34.5 (0.03, 125.3)	705, 26.0 (0.03, 145.3)
Family history of aHUS, *n* (%)						
Yes	2 (10.0)	6 (11.8)	8 (11.3)	34 (7.9)	74 (12.4)	108 (10.5)
No	14 (70.0)	32 (62.7)	46 (64.8)	346 (80.7)	415 (69.5)	761 (74.2)
Missing^b^	0	0	0	0	1 (0.2)	1 (0.1)
Unknown^c^	4 (20.0)	13 (25.5)	17 (23.9)	49 (11.4)	107 (17.9)	156 (15.2)
Timing of MHT, *n* (%)						
Before aHUS	4 (20.0)	4 (7.8)	8 (11.3)	N/A	N/A	N/A
After aHUS	2 (10.0)	7 (13.7)	9 (12.7)	N/A	N/A	N/A
Around the same time	13 (65.0)	36 (70.6)	49 (69.0)	N/A	N/A	N/A
Unknown	1 (5.0)	4 (7.8)	5 (7.0)	N/A	N/A	N/A
New extra-renal manifestations not present at time of initial diagnosis, *n* (%)						
Cardiovascular	6 (30.0)	14 (27.5)	20 (28.2)	77 (17.9)	118 (19.8)	195 (19)
Pulmonary	4 (20.0)	5 (9.8)	9 (12.7)	38 (8.9)	62 (10.4)	100 (9.7)
Central nervous system	5 (25.0)	9 (17.6)	14 (19.7)	70 (16.3)	104 (17.4)	174 (17.0)
Gastrointestinal	4 (20.0)	12 (23.5)	16 (22.5)	84 (19.6)	118 (19.8)	202 (19.7)
Genetics, *n* (%)						
Patients not tested for any variants^d^	5 (25.0)	5 (9.8)	10 (14.1)	116 (27.0)	168 (28.1)	284 (27.7)
Patients tested for at least one variant	15 (75.0)	46 (90.2)	61 (85.9)	313 (73.0)	429 (71.9)	742 (72.3)
Patients tested for at least one variant with a recorded result	7 (35.0)	27 (52.9)	34 (47.9)	120 (28.0)	180 (30.2)	300 (29.2)
Patients tested for anti-CFH-antibodies with a positive result	2 (10.0)	9 (17.6)	11 (15.5)	35 (8.2)	56 (9.4)	91 (8.9)
Patients tested for anti-CFH-antibodies with a negative result	0	0	0	0	0	0
Any variant found or anti-CFH-antibody positive^e^	7 (35.0)	33 (64.7)	40 (56.3)	152 (35.4)	230 (38.5)	382 (37.2)
Triggering conditions, *n* (%)						
Drug-induced aHUS	0	3 (5.9)	3 (4.2)	19 (4.4)	19 (3.2)	38 (3.7)
* Streptococcus pneumoniae* infection	0	1 (2.0)	1 (1.4)	2 (0.5)	8 (1.3)	10 (1.0)
Bone marrow transplant	0	0	0	7 (1.6)	1 (0.2)	8 (0.8)
Autoimmune disease	0	1 (2.0)	1 (1.4)	15 (3.5)	13 (2.2)	28 (2.7)
Drug-induced and autoimmune disease	0	1 (2.0)	1 (1.4)	0	0	0
Patients with kidney transplant, *n* (%)						
Yes	0	33 (64.7)	33 (46.5)	20 (4.7)	304 (50.9)	324 (31.6)
No	18 (90.0)	18 (35.3)	36 (50.7)	386 (90.0)	287 (48.1)	673 (65.6)
Missing	2 (10.0)	0	2 (2.8)	23 (5.4)	6 (1.0)	29 (2.8)
Baseline laboratory parameters,^f^ *n*/*N*; median (min, max)						
Serum creatinine, µmol/L	14/20; 456 (27, 1221)	4/51; 153 (21, 317)	18/71; 260 (21, 1221)	256/429; 240 (18, 1315)	102/597; 233 (21, 1492)	358/1026; 240 (18, 1492)
Lactate dehydrogenase, U/L	13/20; 956 (263, 3332)	8/51; 425 (280, 606)	21/71; 486 (263, 3332)	257/429; 692 (137, 5632)	163/597; 439 (151, 5404)	420/1026; 551 (137, 5632)
Platelet counts, × 10^3^/µL	10/20; 69 (22, 432)	7/51; 185 (117, 455)	17/71; 118 (22, 455)	237/429; 87 (10, 506)	148/597; 142 (11, 618)	385/1026; 111 (10, 618)

*aHUS* atypical hemolytic uremic syndrome, *CFH* complement factor H, *MHT* malignant hypertension

^a^Patients not treated with eculizumab included any patients who were never treated with eculizumab; who received eculizumab after reaching ESKD (defined as kidney transplantation or chronic maintenance dialysis); or who received eculizumab up-to and including one month prior to kidney transplantation

^b^Patients with missing data had no recorded data available within the registry database

^c^Patients with unknown family history had a specific ‘unknown’ data entry in the registry database, based upon clinician input via the recording form

^d^Also includes any patients with missing data

^e^Data is included only for patients who were tested and had a result recorded in the registry database, patients who were tested but had no available results were excluded

^f^Baseline laboratory parameters reported were the closest value to aHUS onset date in either direction

A greater proportion of patients with aHUS and MHT received a kidney transplant compared to patients without MHT (33 [47%] vs 324 [32%]). However, the proportion of patients with aHUS requiring a kidney transplant was higher in those not treated than in those treated with eculizumab, regardless of whether they had comorbid MHT or not (with MHT, 33 [65%] vs 0 [0%]; without MHT, 304 [51%] vs 20 [5%]). Of the 33 not-treated patients with aHUS and MHT who required a kidney transplant, two had a transplant at the time of aHUS diagnosis, two prior to a diagnosis of aHUS, and two had no date of transplantation recorded in the registry; all other patients had a transplant after receiving a diagnosis of aHUS.

Aside from patients with aHUS and MHT treated with eculizumab—who reported no triggering conditions other than MHT—similar, small proportions of patients reported triggering conditions other than MHT in all other patient cohorts (Table 1).

### Time to ESKD or death

**Fig. 2:**
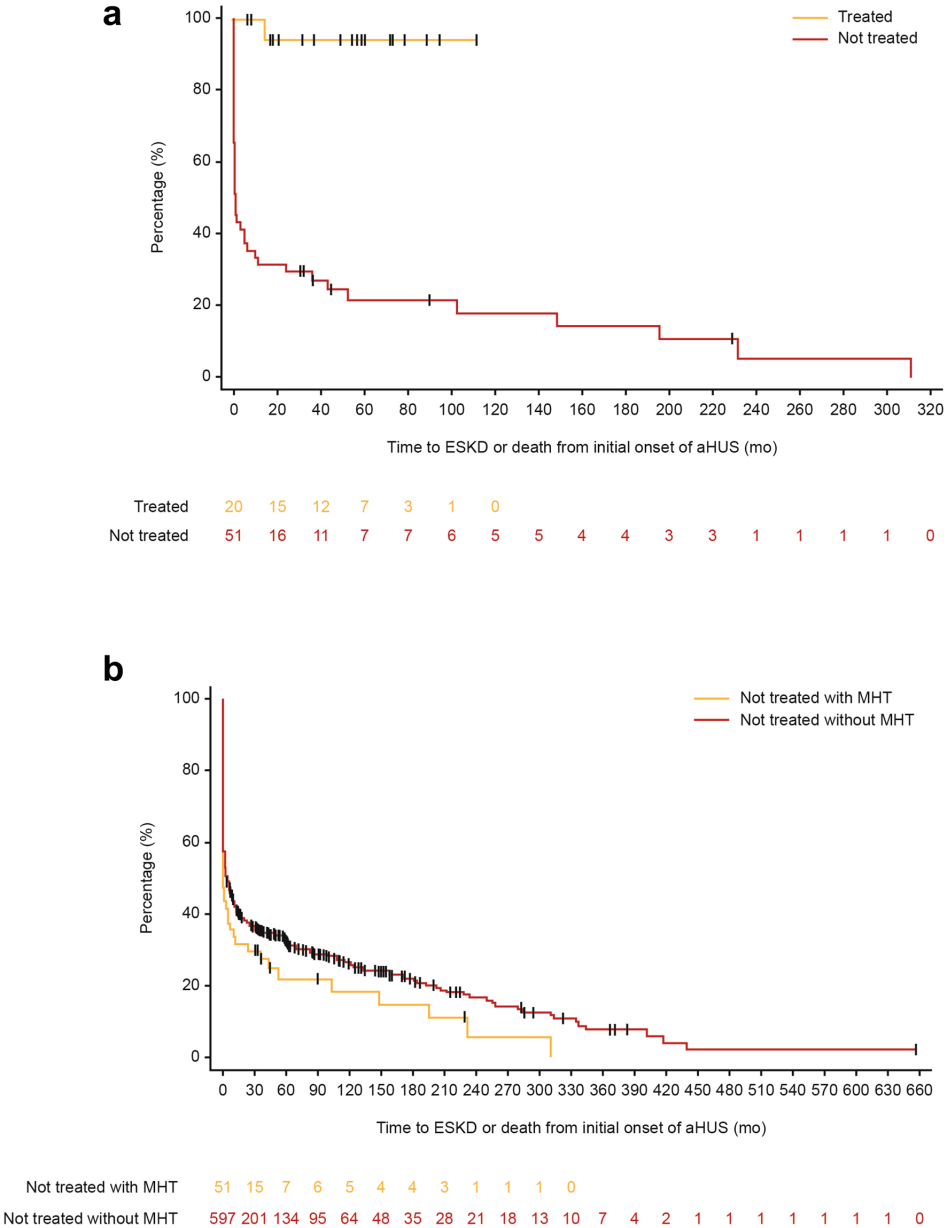
Kaplan–Meier plots for time to ESKD or death from initial onset of aHUS for **a** patients with aHUS and MHT who were treated vs patients with aHUS and MHT who were not-treated; **b** untreated patients with aHUS and MHT vs untreated patients with aHUS without MHT^a^. ^a^71 subjects with ESKD prior to initial onset of aHUS were excluded from this analysis. *aHUS* atypical hemolytic uremic syndrome, *ESKD* end-stage kidney disease, *HR* hazard ratio, *MHT* malignant hypertension, *mo* month, *PE* plasma exchange, *PI* plasma infusion, *TMA* thrombotic microangiopathy

Kaplan–Meier plots and HRs for the combined endpoint of ESKD or death are presented in Fig. 2, and full HR analyses are available in Supplementary Table S2. Figure 2a presents a comparison of treated and not treated patients with aHUS and MHT. Treated patients had a significantly reduced risk of reaching ESKD or death compared to not-treated patients (unadjusted HR [95% CI], 0.04 [0.01–0.30], *P* = 0.002; adjusted HR [95% CI], 0.11 [0.01–0.87], *P* = 0.036). Figure 2b presents a comparison of patients with aHUS both with and without MHT who were not treated with eculizumab. Not-treated patients with aHUS and MHT were not at a significantly increased risk of ESKD or death compared to not-treated patients without MHT (unadjusted HR [95% CI], 1.18 [0.82–1.68], *P* = 0.373; adjusted HR [95% CI], 1.15 [0.80–1.64], *P* = 0.451).

**Fig. 3:**
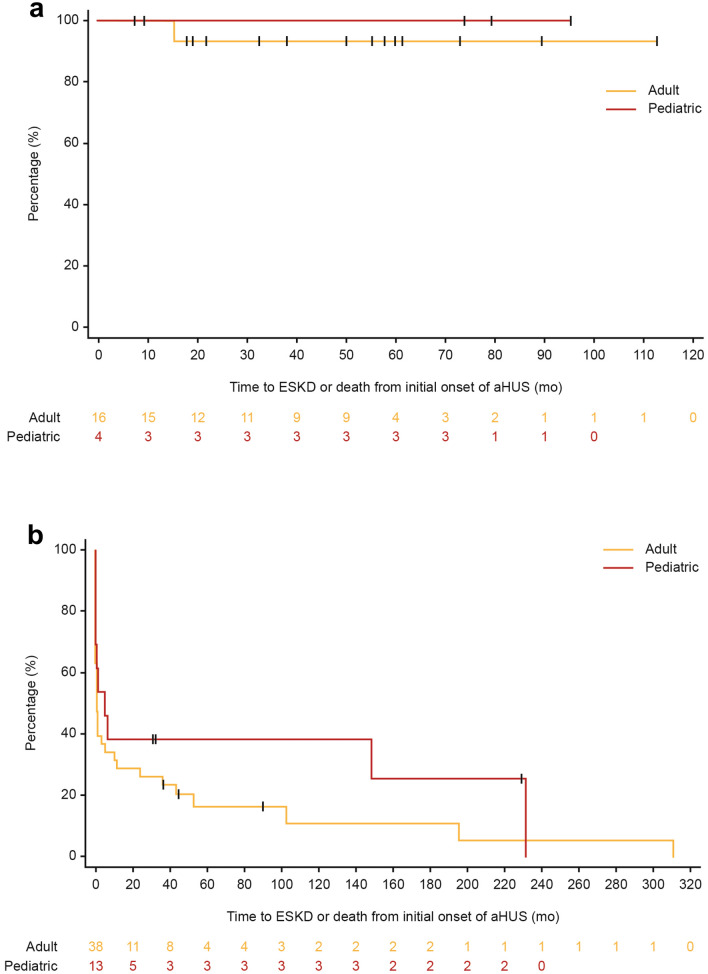
Kaplan–Meier plots for time to ESKD or death from initial onset of aHUS for adult and pediatric patients with aHUS and MHT who were **a** treated with eculizumab or **b** not treated with eculizumab. *aHUS* atypical hemolytic uremic syndrome, *ESKD* end-stage kidney disease, *MHT* malignant hypertension, *mo* month

Kaplan–Meier plots for the combined endpoint of ESKD or death in patients with aHUS and MHT, stratified by age groups (adult or pediatric), are presented in Fig. 3. Figure 3a presents a comparison of adult and pediatric patients with aHUS and MHT who were treated with eculizumab, while Fig. 3b presents a comparison of adult and pediatric patients with aHUS and MHT who were not treated with eculizumab. Adult patients were at greater risk of ESKD or death than pediatric patients, and not-treated patients had worse outcomes than treated patients in both age groups.

## Discussion

This study presents data from the largest comparison of patients with aHUS with and without comorbid MHT to date. In the study population, MHT was reported as occurring at the same time as aHUS symptoms in ~ 2/3 of patients presenting with comorbid aHUS and MHT, irrespective of treatment status, and more patients with aHUS and MHT possessed pathogenic genetic variants or anti-CFH antibodies than patients with aHUS alone (40 [56%] vs 382 [37%]). Further, a much higher proportion of non-treated patients with aHUS and MHT had pathogenic genetic variants or anti-CFH antibodies (33 [65%]) compared to their treated counterparts (7 [35%]). Considering these data, and that these patients were diagnosed with aHUS, it is perhaps surprising that 51 (72%) patients with aHUS and MHT were not treated with eculizumab. However, this may partially be explained by 23 (32%) patients with aHUS and MHT and 323 (31%) patients with aHUS without MHT having a recorded onset of aHUS prior to eculizumab obtaining marketing authorization in 2011. Other possible explanations include 20 (61%) of the 33 not-treated patients with aHUS and MHT who required a kidney transplant reaching ESKD (a criterion for designating patients as not-treated in this study) prior to eculizumab availability, and some patients may also have been treated with eculizumab post-ESKD (another criterion for not-treated designation in this study). Furthermore, while eculizumab treatment status itself is not directly related to the prevalence of pathogenic genetic variants or anti-CFH antibodies, the results suggest that many of the patients listed as not-treated may either have reached ESKD before eculizumab became available or were not initially identified as patients with aHUS prior to ESKD. Indeed, diagnosis of aHUS may often occur late in the disease course, following TMA recurrence, a requirement for long-term dialysis, or kidney transplantation [13]. It is important to note, however, that this study only reports genetic analyses in patients who were screened and had a result reported in the registry; some patients were recorded as having been screened but no subsequent results were reported.

When the combined outcome of time to ESKD or death was assessed, both uni- and multi-variable analyses showed that significantly fewer patients with aHUS and MHT who were treated with eculizumab reached the composite endpoint, compared to not-treated patients. Further, the multi-variable analyses also highlighted that patients who presented with pathogenic genetic variants and/or anti-CFH antibodies, and patients who were adults at the time of aHUS onset, were generally at a higher risk of ESKD or death. However, many other clinical features were similar between these patient groups. As anticipated, patients from both age groups who were not-treated had worse outcomes than their treated counterparts. These results, combined with higher proportions of pathogenic genetic variants and kidney transplants in patients with aHUS and MHT—particularly those not treated with eculizumab—reiterate the importance of establishing an early and accurate diagnosis, as treating the correct patients with C5 inhibitors has been shown to substantially reduce morbidity and mortality [24–27]. However, in this analysis, fewer patients with aHUS and MHT were treated with eculizumab than patients without MHT, despite the potentially counter-intuitive increased incidence of complement gene variants in this patient population. This raises the possibility that clinicians may be continuing to regard TMA as secondary to MHT and proceed with MHT-specific treatment regimens, without considering this as a potential presentation/manifestation of aHUS/CM-TMA [9, 13, 16, 28].

One patient who presented with aHUS and MHT and was treated with eculizumab progressed to ESKD. This patient began treatment with eculizumab in September 2018 and reached ESKD in October 2019, with an interval between initiation and ESKD of 13.01 months.

In their review, Fakhouri and Frémaux-Bacchi stated that while aHUS remains, globally, a rare cause of MHT, MHT frequently complicates aHUS disease course, adding that genetic screening may not be suitable for diagnosis of aHUS as not all patients carry complement gene variants [13]. However, they commented that TMA rarely complicates the course of MHT (5–15% of cases), with the low prevalence limiting assessments of complement gene variants in patients with comorbid severe hypertension and TMA [13]. Our data are therefore important as all patients in the current analysis were diagnosed with both MHT and aHUS/TMA and were seen to have a greater prevalence of pathogenic genetic variants or anti-CFH antibodies. These results suggest that clinicians should explicitly consider genetic screening in this specific patient population. Also, while our results agree with Fakhouri and Frémaux-Bacchi that patients with aHUS can often present with MHT [13], they further suggest that a differential diagnosis of aHUS/CM-TMA should be considered in patients presenting with both MHT and TMA. This is particularly important as, in our analysis, patients with aHUS responded well to eculizumab in the presence of MHT, making an early and correct diagnosis integral to improving patient outcomes [24–27, 29]. This agrees with the paper by Karoui et al.*,* who found that the 5-year renal survival rate was substantially lower in patients with aHUS with identified complement variants and/or hypertensive emergency than their counterparts without these complicating factors [30].

There are several potential limitations to this study—mainly in relation to the nature of registry-derived data, as previously described [23]—leading to missing/incomplete data, particularly around the recording of dates, genetic screening results, blood pressure measurements, and concomitant medication. Specifically relating to blood pressure, these data were not necessarily recorded at the time of MHT and had large variances, making conclusions difficult. Furthermore, the Global aHUS Registry only collects data on patients with a local clinical diagnosis of aHUS (not a centrally defined diagnosis) which may potentially limit the generalizability of these findings to proven CM-TMA populations. While the lack of a central definition of MHT may be a potential limitation of this study, the general clinical characteristics of MHT used for diagnosis are easily assessable and well defined.

This analysis of patients with aHUS and MHT using data from the Global aHUS Registry shows a higher prevalence of pathogenic complement variants or anti-CFH antibodies, alongside a high proportion of kidney transplantation, in patients with aHUS and MHT (particularly in not-treated patients) indicating a potential lack of early/correct diagnosis and high severity of disease in these patients when left untreated. Indeed, patients who were positive for pathogenic variants or anti-CFH antibodies were at greater risk of ESKD or death than patients without them. However, in not-treated patients with aHUS, the concurrent presence of MHT did not appear to significantly impact the risk of reaching ESKD or death, compared to not-treated patients without MHT. Moreover, MHT did not appear to affect the effectiveness of eculizumab, or baseline demographics and characteristics, compared to patients without MHT, although no formal statistical assessment of this comparison was conducted. This study also demonstrates that while clinical characteristics in patients with aHUS and MHT are similar in both pediatric and adult patients, with comparable demographics and baseline clinical measures, patients who were adults at the time of aHUS onset were at greater risk of ESKD or death than patients who were below 18 years of age at the time of aHUS onset. Lastly, the significant difference in the composite endpoint of ESKD or death between patients who were treated with complement C5 inhibition and those who were not-treated highlights the importance of an early and accurate diagnosis in these patients, to allow for the correct use of these therapeutics. Alongside a reiteration of the importance of complement C5 inhibitor therapy in patients with aHUS, the results of this study provide evidence that, in patients presenting with MHT and comorbid TMA, complement genetic screening and consideration of a differential diagnosis of aHUS are warranted to allow for prompt and correct treatment decisions.

## Supplementary Information

Supplementary information is available on the Journal of Nephrology’s website.

Supplementary file1 (PDF 300 KB)

Supplementary file2 (DOCX 95 KB)

## Data Availability

Alexion will consider requests for disclosure of clinical study participant-level data provided that participant privacy is assured through methods like data de-identification, pseudonymization, or anonymization (as required by applicable law), and if such disclosure was included in the relevant study informed consent form or similar documentation. Qualified academic investigators may request participant-level clinical data and supporting documents (statistical analysis plan and protocol) pertaining to Alexion-sponsored studies. Further details regarding data availability and instructions for requesting information are available in the Alexion Clinical Trials Disclosure and Transparency Policy at https://alexion.com/our-research/research-and-development. Link to Data Request Form: https://alexion.com/contact-alexion/medical-information.
