# Supplementary material for: Clinical characteristics and outcomes of a patient population with atypical hemolytic uremic syndrome and malignant hypertension: analysis from the Global aHUS registry
Source: J Nephrol. 2022 Sep 24;36(3):817–28. doi: 10.1007/s40620-022-01465-z (PMC10090001; doi:10.1007/s40620-022-01465-z)
Supplement: Supplementary file 1 — Supplementary file1 (PDF 300 KB) [file 40620_2022_1465_MOESM1_ESM.pdf]

Clinical characteristics and outcomes of a patient population with atypical hemolytic uremic syndrome and malignant hypertension: analysis from the Global aHUS Registry

This is a summary of a clinical study which looked at the differences and similarities between patients who only had atypical hemolytic uremic syndrome (aHUS) and patients who had aHUS and malignant hypertension (MHT). The study also looked at what happened when some of the patients were treated with eculizumab (SOLIRIS®).

- SOLIRIS® was approved to treat aHUS in 2011 in the EU and the USA based on clinical trials which showed that it was effective for treating both children and adults with aHUS.

What is aHUS?

- aHUS is a rare condition caused by problems in a part of the immune system called the complement system. These problems are often caused by genetic mutations. In aHUS, the complement system becomes overactive, which can cause damage to red blood cells and lead to clots forming in small blood vessels. This results in a condition known as **thrombotic microangiopathy**, or **TMA**. TMA results in damage to organs such as the kidneys by stopping blood from reaching them.

- SOLIRIS® is usually prescribed for patients diagnosed with aHUS to reduce overactivity of the complement system. It reduces red blood cell damage and clot formation. Patients with aHUS who are not treated correctly or quickly enough are at risk of kidney failure (which may mean they need a kidney transplant) and damage to other organs (called extrarenal complications), and/or death.

- **MHT** is a rare and severe form of high blood pressure (hypertension). Like aHUS, MHT can also cause TMA and organ damage. Patients with MHT are usually treated with medications to lower their blood pressure. Some patients with aHUS can also have MHT.

Why is this important?

- aHUS and MHT can have similar symptoms and some patients may have both conditions at the same time. However, they are treated differently, so it is important to diagnose and treat these conditions correctly to make sure that the risks of poor outcomes are reduced.

What did this study investigate?

- This study used data from the Global aHUS Registry (ClinicalTrials.gov Identifier: NCT01522183), which is the largest collection of information on patients with aHUS. Information was collected on patients with aHUS who had or did not have MHT at the same time. Information was also collected on patients who were or were not treated with SOLIRIS®. The study looked at factors including age, race, sex, and whether patients had received a kidney transplant. It also looked at whether patients had genetic mutations affecting parts of the complement system or antibodies against the complement system (**anti-complement antibodies**).

What did this study find?

Total patients (aHUS with/without MHT)

N=1097

aHUS with MHT	aHUS without MHT
n=71	n=1026
Treated* (n=20)	Treated* (n=429)
Not-treated* (n=51)	Not-treated* (n=597)

	aHUS		Treated*		Not treated*	
	With MHT	vs Without MHT	With MHT	vs Without MHT	With MHT	vs Without MHT
Received kidney transplantation	47%	32%	0%	5%	65%	51%
Genetic mutation or anti-complement antibodies	56%	37%	35%	35%	65%	39%

*SOLIRIS®

What are the main conclusions of this study?

- Patients with MHT were more likely to have received a kidney transplant and/or have genetic mutations or anti-complement antibodies than those without MHT. The results show that aHUS is a very severe illness and that patients with this condition often have poor outcomes, particularly if not treated promptly and correctly.
- The risk of end-stage kidney disease (ESKD) or death was significantly lower in treated patients with aHUS and MHT than in not-treated patients, showing that SOLIRIS® is effective in patients with both aHUS and MHT.
- Because aHUS and MHT can have similar symptoms, it can sometimes be difficult to tell them apart. Also, as this study shows, some patients can have aHUS and MHT at the same time, making diagnosis and treatment choices even more difficult. These results show how important it is to diagnose patients quickly so that the right treatment is given, and the risks of poor outcomes are reduced.
- This summary is based on the research article by Dr Jean-Michel Halimi and colleagues, entitled: *Clinical characteristics and outcomes of a patient population with atypical hemolytic uremic syndrome and malignant hypertension: analysis from the Global aHUS Registry*.

Acknowledgements

- Alexion, AstraZeneca Rare Disease, Boston, MA sponsored this study. Alexion and the investigators thank the patients and their families for their participation in, and support for, this clinical study. The authors also thank Global aHUS Registry investigators who contributed data, Scientific Advisory Board members of the Global aHUS Registry and National Coordinators of the Global aHUS Registry. The authors of the original research article reviewed and approved the summary.

Glossary

Anti-complement antibodies: The presence of antibodies against complement system factors (i.e., complement factor H) which can cause problems with the complement system, such as overactivation.

Eculizumab (SOLIRIS®): A drug developed by Alexion, AstraZeneca Rare Disease approved in the United States and in the EU in 2011 for the treatment of aHUS.

End-stage kidney disease (ESKD): End-stage kidney disease, or kidney failure, occurs when the kidneys no longer work as they should and cannot clear waste from the blood.

Thrombotic Microangiopathy (TMA): Formation of numerous small blood clots in the smaller blood vessels of the body, causing substantial damage to the vessel walls which often results in severe organ damage.

Malignant hypertension (MHT): A severe form of high blood pressure (hypertension) that can cause TMA and, in some cases, organ damage.
