# Supplementary material for: Clinical characteristics and outcomes of a patient population with atypical hemolytic uremic syndrome and malignant hypertension: analysis from the Global aHUS registry
Source: J Nephrol. 2022 Sep 24;36(3):817–28. doi: 10.1007/s40620-022-01465-z (PMC10090001; doi:10.1007/s40620-022-01465-z)
Supplement: Supplementary file 2 — Supplementary file2 (DOCX 95 KB) [file 40620_2022_1465_MOESM2_ESM.docx]

**Supplementary information**

**Supplementary Table S1 | Patient demographics stratified by presence or absence of comorbid MHT, treatment status and adult vs pediatric designation**

| **Characteristics** | **aHUS with MHT (n = 71)** | | | | **aHUS without MHT (n = 1026)** | | | |
| --- | --- | --- | --- | --- | --- | --- | --- | --- |
|  | **Treated (n = 20)** | | **Not treated (n = 51)^a^** | | **Treated (n = 429)** | | **Not treated (n = 597)^a^** | |
|  | **Adults n = 16** | **Pediatrics**  **n = 4** | **Adults**  **n = 38** | **Pediatrics**  **n = 13** | **Adults**  **n = 283** | **Pediatrics**  **n = 146** | **Adults**  **n = 412** | **Pediatrics**  **n = 185** |
| Age at aHUS diagnosis [years], median (min, max) | 35.15  (24.2, 63.1) | 0.81  (0.5, 4.0) | 32.92  (18.6, 68.2) | 6.69  (0.7, 17.0) | 40.83  (18.4, 90.6) | 4.02  (0.02, 17.89) | 39.69  (18.2, 84.2) | 3.80  (0.003, 41.25) |
| Sex [female], n (%) | 9 (56.3) | 2 (50) | 21 (55.3) | 11 (84.6) | 205 (72.4) | 74 (50.7) | 267 (64.8) | 78 (42.2) |
| Total duration of eculizumab treatment [months], n, median (mix, max) | 16, 26.6 (6.0, 67.9) | 4, 58.9 (0.2, 71.6) | 24, 34.8 (0.07, 110.9) | 7, 37.1 (17.5, 80.4) | 274, 20.8 (0.03, 125.7) | 145, 22.7 (0.03, 145.3) | 215, 29.2 (0.03, 125.3) | 71, 47.2 (0.1, 121.8) |
| Family history of aHUS, n (%) |  |  |  |  |  |  |  |  |
| Yes | 1 (6.3) | 1 (25.0) | 4 (10.5) | 2 (15.4) | 17 (6.0) | 17 (11.6) | 33 (8.0) | 41 (22.2) |
| No | 11 (68.8) | 3 (75.0) | 23 (60.5) | 9 (69.2) | 224 (79.2) | 122 (83.6) | 284 (68.9) | 131 (70.8) |
| Missing^b^ | 0 | 0 | 0 | 0 | 0 | 0 | 94 (22.8) | 0 |
| Unknown^c^ | 4 (25.0) | 0 | 11 (28.9) | 2 (15.4) | 42 (14.8) | 7 (4.7) | 1 (0.2) | 13 (7.0) |
| Timing of MHT, n (%) |  |  |  |  |  |  |  |  |
| Before aHUS | 4 (25.0) | 0 | 4 (10.5) | 0 | N/A | N/A | N/A | N/A |
| After aHUS | 1 (6.3) | 1 (25.0) | 6 (15.8) | 1 (7.7) | N/A | N/A | N/A | N/A |
| Around the same time | 10 (62.5) | 3 (75.0) | 24 (63.2) | 12 (92.3) | N/A | N/A | N/A | N/A |
| Unknown | 1 (6.3) | 0 | 4 (10.5) | 0 | N/A | N/A | N/A | N/A |
| New extra-renal manifestations not present at time of initial diagnosis, n (%) |  |  |  |  |  |  |  |  |
| Cardiovascular | 3 (18.8) | 3 (75.0) | 8 (21.1) | 6 (46.2) | 57 (20.1) | 20 (13.7) | 83 (20.1) | 35 (18.9) |
| Pulmonary | 3 (18.8) | 1 (25.0) | 4 (10.5) | 1 (7.7) | 29 (10.2) | 9 (6.2) | 44 (10.7) | 18 (9.7) |
| Central nervous system | 2 (12.5) | 3 (75.0) | 7 (18.4) | 2 (15.4) | 50 (17.7) | 20 (13.7) | 70 (17.0) | 34 (18.4) |
| Gastrointestinal | 3 (18.8) | 1 (25.0) | 9 (23.7) | 3 (23.1) | 57 (20.1) | 27 (18.5) | 81 (19.7) | 37 (20.0) |
| Any variant found or anti-CFH-antibody positive^d^, n (%) | 5 (31.3) | 2 (50.0) | 26 (68.4) | 7 (53.8) | 78 (27.6) | 74 (50.7) | 142 (34.5) | 88 (47.6) |
| Triggering conditions, n (%) |  |  |  |  |  |  |  |  |
| Drug-induced aHUS | 0 | 0 | 3 (7.9) | 0 | 17 (6.0) | 2 (1.4) | 17 (4.1) | 2 (1.1) |
| Streptococcus pneumoniae infection | 0 | 0 | 0 | 1 (7.7) | 1 (0.4) | 1 (0.7) | 1 (0.2) | 8 (4.0) |
| Bone marrow transplant | 0 | 0 | 0 | 0 | 4 (1.4) | 3 (2.1) | 0 | 1 (0.5) |
| Autoimmune disease | 0 | 0 | 1 (2.6) | 0 | 13 (4.6) | 2 (1.4) | 11 (2.7) | 2 (1.0) |
| Drug-induced and autoimmune disease | 0 | 0 | 1 (2.6) | 0 | 0 | 0 | 0 | 0 |
| Patients with kidney transplant, n (%) |  |  |  |  |  |  |  |  |
| Yes | 0 | 0 | 27 (71.1) | 6 (46.2) | 12 (4.2) | 8 (5.5) | 205 (49.8) | 105 (53.0) |
| No | 14 (87.5) | 4 (100) | 11 (28.9) | 7 (53.8) | 255 (90.1) | 131 (89.7) | 203 (49.3) | 91 (46.0) |
| Missing | 2 (12.5) | 0 | 0 | 0 | 16 (5.7) | 7 (4.8) | 4 (1.0) | 2 (1.0) |
| Baseline laboratory parameters^e^, n/N; median (min, max) |  |  |  |  |  |  |  |  |
| Serum creatinine, µmol/L | 13/16; 332.4 (79.6, 1220.8) | 3/4; 125.5 (26.5, 128.5) | 23/38; 235.4 (84.0, 1330.0) | 5/13; 82.0 (21.2, 189.0) | 194/283; 260.8 (18.6, 1315.0) | 101/146; 135.0 (18.0, 789.4) | 272/412; 260.2 (38.0; 1358.0) | 121/185; (88.0 (21.2, 1292.0) |
| Platelet counts, x10^3^/µL | 9/16; 198.0 (22.0, 432.0) | 2/4; 49.5 (36.0, 63.0) | 23/38; 240.0 (56.0, 455.0) | 6/13; 179.5 (84.0, 323.0) | 182/283; 112.5 (10.0, 420.0) | 97/146; 126.0 (11.0, 568.0) | 274/412; 190.0 (17.0, 618.0) | 125/185; 229.0 (29.0, 478.0) |
| Lactate dehydrogenase, U/L | 12/16; 275.0 (172.0, 2295.0) | 3/4; 486.0 (348.0, 3332.0) | 27/38; 260.0 (134.0, 606.0) | 6/13; 315.5 (176.0, 784.0) | 206/283; 430.5 (134.0, 3852.0) | 93/146; 1046.0 (155.7, 5632.0) | 265/412; 290.0 (109.0, 5010.0) | 98/185; 272.0 (122.0, 5404.0) |

^a^Patients not treated with eculizumab included any patients who were never treated with eculizumab; who received eculizumab after reaching ESKD (defined as kidney transplantation or chronic maintenance dialysis); or who received eculizumab up-to and including one month prior to kidney transplantation; ^b^Patients with missing data had no recorded data available within the registry database; ^c^Patients with unknown family history had a specific ‘unknown’ data entry in the registry database, based upon clinician input via the recording form; ^d^Data is included only for patients who were tested and had a result recorded in the registry database, patients who were tested but had no available results were excluded; ^e^Baseline laboratory parameters reported were the closest value to enrolment in the registry in either direction (not treated patients) or the closest value to treatment start date in either direction (treated patients).

aHUS, atypical hemolytic uremic syndrome; CFH, complement factor H; MHT, malignant hypertension.

**Supplementary Table S2 | Hazard ratios for time to ESKD or death from initial onset of aHUS for untreated population – analysis with and without propensity weights**

| **Covariates** | **Statistics (N)** | **with propensity weights** | | | | **without propensity weights** | | | |
| --- | --- | --- | --- | --- | --- | --- | --- | --- | --- |
|  |  | **Unadjusted Hazard Ratio**  **(95% CI)*** | **p-value** | **Adjusted**  **Hazard Ratio****  **(95% CI)** | **p-value** | **Unadjusted Hazard Ratio**  **(95% CI)*** | **p-value** | **Adjusted**  **Hazard Ratio****  **(95% CI)** | **p-value** |
| Without MHT [a] | 338 | --- |  |  | --- | --- |  | --- |  |
| With MHT | 39 | 1.18  (0.82, 1.68) | 0.373 | 1.15  (0.80, 1.64) | 0.451 | 1.25  (0.87, 1.78) | 0.230 | 1.14  (0.79, 1.64) | 0.472 |
| Age at initial onset of AHUS | Pediatric [a] = 137 |  |  |  |  |  |  |  |  |
|  | Adult = 240 |  |  | **1.64  (1.26, 2.13)** | **0.0002** |  |  | **1.69**  **(1.30, 2.18)** | **<0.0001** |
| Sex | Male [a] = 172 |  |  | --- |  |  |  | --- |  |
|  | Female = 205 |  |  | 0.92  (0.72, 1.17) | 0.511 |  |  | 0.91  (0.72, 1.16) | 0.448 |
| Genetic Pathogenic mutation | Tested for at least 5 pathogenic variants  with no mutation identified [a] = 157 |  |  | --- |  |  |  | --- |  |
|  | Any mutation found or Anti CFH-antibody positive = 220 |  |  | **1.30**  **(1.01, 1.67)** | **0.040** |  |  | **1.31**  **(1.03, 1.66)** | **0.028** |

[a] Reference group

Note: 71 subjects with ESKD prior to initial onset of aHUS were excluded from the analysis.

* Not adjusted for covariates

** Adjusted for age at initial onset of aHUS, sex and genetics
